# T60. HEALTH LITERACY IN PEOPLE WITH SCHIZOPHRENIA ATTENDING COMMUNITY MENTAL HEALTH CLINICS

**DOI:** 10.1093/schbul/sby016.336

**Published:** 2018-04-01

**Authors:** Sumana Thomson, Cherrie Galletly, Christopher Prener, Suzanne Garverich, Dennis Liu, Alisa Lincoln

**Affiliations:** 1 The University of Adelaide; 2 Saint Louis University; 3 Northeastern University

## Abstract

**Background:**

Health literacy (HL) has been defined as the degree to which individuals possess the capacity to obtain, process, understand and utilise basic health information. For people with schizophrenia, important aspects of their HL include the ability to understand information about their illness and treatment, taking medications correctly, and interacting with clinicians.

Schizophrenia is associated with lower levels of education, which has been found to negatively impact on HL. Further, schizophrenia is often associated with cognitive impairment, but the relationship between HL and cognitive function in this patient population is not known.

Studies of HL in people with physical disorders have demonstrated that people with poor HL have poorer outcomes, with greater morbidity and mortality. There has been very little research into HL in schizophrenia, although it may be expected that those with poor HL might have more difficulty managing their illness and interacting with clinical services.

**Methods:**

People living in the community, and attending public community mental health outpatient clinics in the Northern suburbs of Adelaide, Australia, were invited to participate in the study. They were interviewed by trained research staff, and completed the WAIS VI digit symbol coding (DSC), Verbal Fluency (VF; animal naming), the short version of the Test of Functional Health Literacy in Adults (S-TOFHLA) along with two parts of the Woodcock-Johnson III measuring aural literacy (Part 4; WJ4) and reading literacy (Part 9; WJ9). Of 101 participants, 62 had schizophrenia, while the other 39 had a range of other diagnoses.

**Results:**

The 62 participants with schizophrenia had a mean age of 41.2 (SD 9.9) years and 61% were male. They had a mean of 11.02 (SD 1.5) years of education. The remaining participants had a mean age of 43.3 (SD 13.4) years, 46% were male, and the mean years of education was 11.3 (SD 2.5).

90% of the schizophrenia group were at or below 8th grade (Year 8) level for aural literacy, and 63% were at or below 8th grade (Year 8) for reading literacy.

Those with a schizophrenia diagnosis had lower scores on the WJ9 (mean 8.3, SD 4.5) compared with the non-schizophrenia group (mean 11, SD 5.1); t = 2:739; p = 0:007, medium Cohen’s D effect size (D = 0:548). However, there was not a significant difference (t = 1.975, p = .051) in aural literacy between the schizophrenia and non-schizophrenia groups.

Using the S-TOFHLA, 81% of the schizophrenia group had adequate HL; 6% were marginal and 13% were inadequate. In contrast, 97% of the non-schizophrenia group had adequate HL. The schizophrenia group had lower mean S-TOFHLA scores (mean 28.6, SD 7.5; compared to mean 31.8, SD 4.8); t = 2.369; p = 0.020, medium Glass’s effect size (G = 0.657).

In all subjects, there was a moderate, positive relationship (r = 0.359; p < .05) between education and the TOFHLA score. There was also a positive correlation between the S-TOFHLA score and the aural and reading literacy scores

**Discussion:**

The majority of people with schizophrenia had very poor aural and verbal literacy, and there was a correlation between education and literacy skills, and HL. People with schizophrenia tend to have less years of school attendance, and cognitive impairment is a core component of the disorder.

However, only 19% of the schizophrenia group had inadequate HL. This finding suggests that their HL skills are better than expected, given their educational and literacy deficits. This may reflect their engagement in case management and psychoeducation whilst attending the community MH clinic; the clinicians might have been providing effective ongoing education about managing their mental illness, medication and interactions with health services.

